# The Toronto older adults gait archive: video and 3D inertial motion capture data of older adults’ walking

**DOI:** 10.1038/s41597-022-01495-z

**Published:** 2022-07-11

**Authors:** Sina Mehdizadeh, Hoda Nabavi, Andrea Sabo, Twinkle Arora, Andrea Iaboni, Babak Taati

**Affiliations:** 1 KITE – Toronto Rehabilitation Institute, University Health Network, Toronto, ON Canada; 2 Institute of Biomedical Engineering, University of Toronto, Toronto, ON Canada; 3 Department of Psychiatry, University of Toronto, Toronto, ON Canada; 4 Department of Computer Science, University of Toronto, Toronto, ON Canada; 5 Vector Institute for Artificial Intelligence, Toronto, ON Canada

**Keywords:** Geriatrics, Biomedical engineering

## Abstract

We introduce the Toronto Older Adults Gait Archive, a gait dataset of 14 older adults containing 2D video recordings, and 2D (video pose tracking algorithms) and 3D (inertial motion capture) joint locations of the lower body. Participants walked for 60 seconds. We also collected participants’ scores on four clinical assessments of gait and balance, namely the Tinneti performance-oriented mobility assessment (POMA-gait and -balance), the Berg balance scale (BBS), and the timed-up-and-go (TUG). Three human pose tracking models (Alphapose, OpenPose, and Detectron) were used to detect body joint positions in 2D video frames and a number of gait parameters were computed using 2D video-based and 3D motion capture data. To show an example usage of our datasets, we performed a correlation analysis between the gait variables and the clinical scores. Our findings revealed that the temporal but not the spatial or variability gait variables from both systems had high correlations to clinical scores. This dataset can be used to evaluate, or to enhance vision-based pose-tracking models to the specifics of older adults’ walking.

## Abstract (summary)

**Table:** 

Measurement(s)	Lower Extremity Gait, CTCAE
Technology Type(s)	digital camera • Accelerometer
Sample Characteristic - Organism	Homo
Sample Characteristic - Environment	nursing home
Sample Characteristic - Location	City of Toronto

## Background & Summary

Falls are a major health problem in older adults and are one of the main causes of injuries and premature death in adults over 65 years old. Approximately one in three older adults fall annually, of whom 24% sustain serious injuries^1^. Most falls occur during walking^2^ and impaired gait is an important cause of falls.

Clinically established techniques for gait monitoring typically require technologies such as motion capture systems which are expensive and time consuming to use, require specialized expertise and staff to operate, and are not widely available for clinical use. As a result, gait monitoring practices have mainly involved cross-sectional gait assessments in laboratory settings or under experimental conditions which do not reflect the cognitive and physical demands of natural walking or usual locomotion^3^.

Techniques based on computer vision technology and human pose estimation in video data can address these limitations. A number of algorithms have been developed for human pose tracking that are capable of automated analysis of human walking using only standard RGB camera videos and deep learning models^4–11^. These packages are freely available and can be used to process videos of human walking in any setting with minimal cost and technical expertise^9^. Gait parameters can subsequently be computed from the sequence of tracked body parts^12^. However, for use in clinical applications, there is a need to validate gait variables calculated from pose tracking data against gold standard methods for measuring human gait data, e.g., three-dimensional (3D) motion capture systems^9^. It is also important to specifically test the validity of the pose tracking algorithms in older adults as their posture and gait are different to that in young adults and are characterized by lower speed and greater variability^13^. Therefore, there is a need for publicly available datasets that contain both video and 3D motion capture files of older adults’ walking so that researchers can validate pose tracking algorithms and models to the specifics of older adults’ gait.

While there are some open datasets of older adult gait videos, they generally suffer from limitations such as few gait cycles, few markers, no raw data, and are not suitable for quantitative gait monitoring for mobility assessment^14–18^. To help with these limitations, we aimed to make our data of older adults’ walking 2D videos and 3D motion capture publicly available. This new dataset will help accelerate scientific advancement in the field of pose estimation, gait monitoring, and fall risk assessment in older adults by encouraging new analyses. To demonstrate an example usage of our dataset, we conducted a correlation analysis between the gait variables from the videos and the clinical gait and balance scores of participants. This analysis will provide evidence for the relationship between the video gait measures and clinically meaningful changes in gait and balance, as measured in the clinical assessments, and thus can contribute to the use of video gait monitoring in clinical settings. In a previous study^19^, we performed a separate analysis of this data and demonstrated that there is a high correlation between the temporal gait variables calculated from the pose-tracking algorithms and those calculated from a gold standard inertial motion capture system.

## Methods

### Participants

Participants were 14 residents (healthy older adults age >65 years; 11 female and 3 male) of a retirement home who consented to participate in the study. The average (standard deviation) age, height, and mass of the participants were 86.7 (6.2) years, 165.6 (9.9) cm, and 64.0 (12.5) kg, respectively. The University of Toronto Ethics Board approved the study protocol. Residents of the retirement home were sent a recruitment letter briefly describing the study, and expressed their interest in participation by calling the research assistant. Participants provided written consent before participating in the study and consented to the public sharing of the study data. The inclusion criteria were being older than 65 years and an ability to walk independently over a distance of 20 meters. The data was pseudonymized and a unique identification number was assigned to each participant. Participants’ demographic details are presented in Table 1.

**Table 1:** Demographic and clinical test scores of the 14 participants.

	Mean (standard deviation)	Range
Age (years)	86.7 (6.2)	75–99
Number of men (%)	3 (21.4)	
Weight (kg)	63.9 (12.5)	48.1–81.6
Height (cm)	165.6 (10.0)	147.3–182.8
Number of people with falls in past 6 months (%)	2 (14.2)	
POMA-balance	12.8 (1.6)	9–14
POMA-gait	11.7 (0.6)	10–12
TUG (s)	12.2 (4.2)	8.3–25
BBS	45.0 (6.3)	34–53

POMA = Tinneti performance-oriented mobility assessment; TUG = time up-and-go, BBS = Berg balance scale.

### Protocol

The experimental protocol consisted of two parts, the clinical tests (which were not video recorded) and the walking task (which was recorded both by the cameras and the 3D inertial motion capture unit (IMU) system). All tests were competed in an independent retirement home.

### Clinical tests

We measured the participant’s age, height, weight, and fall history in the last 6 months. The following clinical tests were performed by the research assistant: the Mini-Mental State Examination (MMSE^20^), the Tinneti performance-oriented mobility assessment (POMA) for balance and gait^21^, the Berg balance scale (BBS)^22^), and timed-up-and-go (TUG^23^).

### Walking task

After completion of the clinical tests, participants walked back and forth for one minute along the long axis of a large room. All participants started the test by walking towards the camera (towards the negative X axis, see below). The walking distance was approximately 13 meters and the walking surface was flat. Participants were instructed to walk at their normal pace with the ‘go’ signal from the assessor and to stop walking with the ‘stop’ signal. A clinical assistant walked beside the participants at a safe distance but did not provide any pacing or physical support. For each participant, a calibration protocol according to the instruction of the IMU system (Xsens) manual was completed before participating in the walking task. This was repeated until an accepted calibration quality was provided by the Xsens system.

### The 3D inertial motion capture system

An Xsens MVN Awinda system (Xsens, Enschede, Netherlands) comprising of seven wireless inertial measurement units, a receiver hub and straps was used to record participants’ walking at 100 Hz. The seven lower body Xsens sensors were attached to the right and left feet, shanks, thighs and the sacrum. For all segments, the long axis of the sensor was aligned with the long axis of the segment. The MVN Analyze software (Xsens, Enschede, Netherlands) was used to record the walking tasks. The global coordinate system was oriented such that the positive X coordinate was approximately along the direction of walking, the Y coordinate was approximately along the body medial or lateral (depending on the direction of motion) and Z pointed upward.

### Cameras

**Fig. 1:**
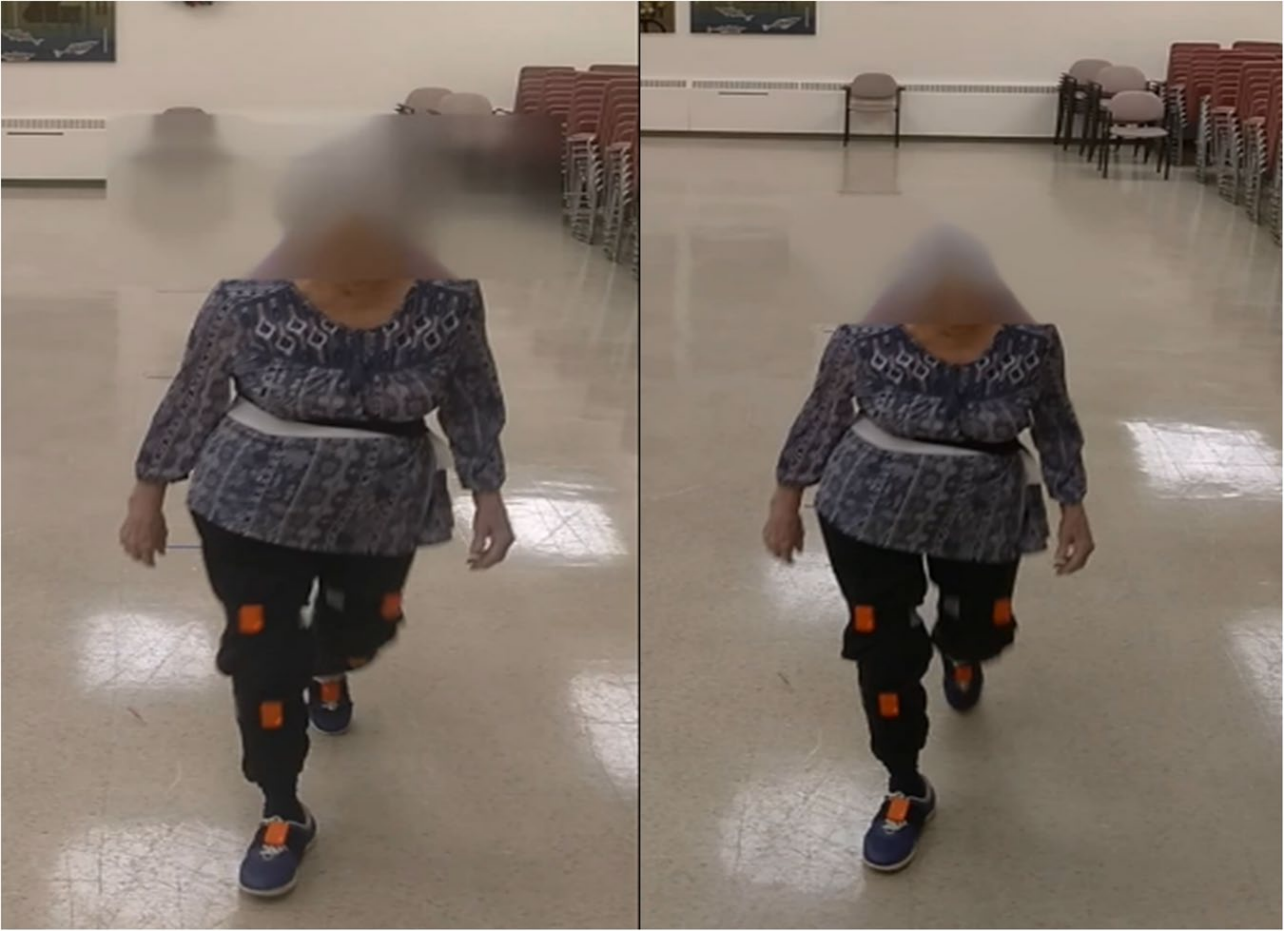
An example screenshots of a participant’s walking as seen from the bottom (left) and top (right) camera. The inertial sensors are attached to the right and left lower body segments. The participant’s face is blurred to protect her identity.

Two Motorola Moto G5 Play cell phones (Motorola, Chicago, IL), equipped with a 13 mega pixel back camera capable of recording videos at 30 frame per second at 1080p resolution, were used to record the walking videos. These cameras were placed at two different heights of 111 cm (approximately eye-level) and 205 cm, chosen to mimic wall-mounted (straight) and ceiling-mounted (tilted down) camera viewing angles. Cameras were pointed towards the positive X of the global coordinate system (Fig. 1). Note that no synchronization between the camera and Xsens system were done in this study.

### Video pose tracking

The recorded videos were first cropped temporally, selecting only the sections of the recordings when the participant was continuously walking towards (front view) or away (back view) from the camera. Three open-source human pose estimation libraries i.e., Alphapose^10^ (YOLOv3-spp detector, pretrained ResNet-50 backbone), OpenPose^4^ (Windows demo release 1.5.1), and Detectron^11^ (R-CNN R101-FPN pretrained model backbone) were then used to extract the joint positions in each frame of the cropped videos. They were pretrained with default settings. OpenPose uses a bottom-up approach that first predicts part affinity fields (PAFs) and confidence maps of joints. The PAFs are subsequently used to perform part association and prediction of overall body poses. Conversely, Detectron and AlphaPose both implement top-down designs. Detectron employs an architecture that simultaneously predicts bounding boxes, segments, and joint keypoints. AlphaPose uses a sequential architecture that first places bounding boxes around each person in the image and then performs keypoint prediction within the bounding box. The pose estimation models provide the lateral and vertical positions (in the frontal view) of body joints in each frame of the input video, as well as a score representing the model’s confidence in its prediction of the joint position. The predicted joint positions in each frame were aggregated temporally to obtain joint trajectories of the participant’s movement in each video. The confidence scores provided by the pose estimation libraries were used to identify and remove and linearly interpolate joint positions at time steps where the pose estimation library predicted a joint with low confidence. As the confidence scores output from the different pose estimation libraries are not calibrated, the threshold used to denote low confidence varied for each library (0.5 for Alphapose, 0.3 for OpenPose, and 0.15 for Detectron). These threshold values were chosen by trial and error to make sure that the keypoints in less than 10 percent of the frames were missing or had low confidence. The pose estimation libraries sometimes erroneously labeled a joint on the left side of the body as the corresponding joint on the right side. To address this, the joint trajectories were visually inspected and these errors were manually corrected. Finally, a zero-lag second-order low-pass Butterworth filter with a cut-off frequency of 8 Hz was used to temporally smooth the joint positions^24^.

### Gait variables

Gait variables were calculated for the recorded gait from the two systems, i.e. i) using the 3D joint coordinates extracted using the MVN Analyze (Xsens, Enschede, Netherlands), and ii) by processing the recorded color videos via the Alphapose pose tracking algorithm to obtain 2D joint (pixel) coordinate. Spatial gait variables (e.g. step width, and estimated margin of stability) calculated from the video were normalized by hip width to account for the perspective. Gait variables used were cadence (steps per minutes), step time and step width and their variability (coefficient of variation, CV), and estimated margin of stability (eMOS, which is the distance between velocity–corrected center of mass from the foot at stance in the lateral direction). The details of calculating these variables are presented in our previous papers^12,25–27^.

### Correlation analysis

To demonstrate an example use of our dataset, we calculated the correlation between the gait variables (calculated from both the 3D motion capture system and from the videos using the Alphapose algorithm) and the scores of the clinical gait and balance assessments (i.e. POMA-gait, POMA-balance, TUG, and BBS).

### Statistical analysis

Pearson’s correlation coefficients (R) were used to determine the correlation between the gait variables calculated from the video and from the Xsens system and the clinical gait and balance scores. The correlation coefficients (R) and p-values were reported. The significance level was 0.05. We have chosen not to apply any mathematical corrections for multiple comparisons. This is because these analyses are primarily descriptive in nature and are not testing any specific hypotheses, but rather are hypothesis-generating. In the balance, we prefer to risk a Type I error than a Type II error. By leaving the results uncorrected, this allows the reader to account for the multiple comparisons when interpreting the results, rather than including them within the calculations.

## Data Records

The initial plan was to recruit 20 participants; however the recruitment ended after 14 participants due to the Covid-19 pandemic (mean age = 86.7, Table 1). Out of the 14 participants who took part in the study, data for three were not suitable for analysis (their Xsens data had calibration problems). The videos, clinical scores, and results of the pose tracking of these three participants are included in the dataset but not included in the analyses, leaving the data of 11 participants for the analysis (mean age = 86.5). The average values of the six gait measures for the Xsens system and the pose tracking algorithm are presented in Table 2.

**Table 2:** Average (standard deviation) of the gait variables calculated from the motion capture and videos.

Gait variable	Motion capture		Camera (using Alphapose tracking)			
	Front view walks	Back view walks	Front view walks		Back view walks	
			Top camera	Bottom camera	Top camera	Bottom camera
Number of steps (for 11 participants)	373	241	373	373	241	241
Cadence (steps per minute)	105.22 (9.09)	105.00 (10.33)	104.27 (10.55)	107.06 (9.79)	105.71 (11.03)	105.58 (11.06)
Step time (s)	0.55 (0.05)	0.58 (0.06)	0.58 (0.08)	0.58 (0.06)	0.57 (0.07)	0.57 (0.07)
Step width (m)	0.10 (0.04)	0.10 (0.04)	0.63 (0.10)	0.62 (0.10)	0.54 (0.09)	0.53 (0.08)
CV step time	0.07 (0.03)	0.07 (0.04)	0.14 (0.06)	0.07 (0.03)	0.18 (0.14)	0.09 (0.09)
CV step width	0.41 (0.18)	0.43 (0.19)	0.28 (0.08)	0.31 (0.09)	0.28 (0.09)	0.29 (0.08)
MOS (m)	0.05 (0.02)	0.05 (0.02)	0.25 (0.04)	0.23 (0.05)	0.22 (0.03)	0.21 (0.03)

^*^ Except for cadence and step time, the values calculated from the pose tracking algorithms are dimensionless (normalized by hip width) and cannot be directly compared with motion capture values.

### Dataset

The files containing the raw data as well as the codes for data analysis are available at Figshare.com^28^. It includes three folders, namely Xsens, Pose tracking, and Videos. The Xsens folder contains the comma separated value (CSV) files of 3D global coordinates of the body landmarks (in meters) recorded and calculated using the Xsens system. It also contains the.bvh file format created by the Xsens MVN Animate software. The Xsens CSV files are labelled as “OAWXX” where “OAW” stands for “Older Adults Walking” and XX is the participant’s number. In these files, the first two columns are the frame numbers and timestamps, and the remaining 27 columns contain the 3D global coordinates of the lower body joints.

The pose tracking folder contains the CSV files of the joint positions (in pixels) in each frame of the cropped videos separated by the pose tracking libraries used (Alphapose, Detectron, and OpenPose) and the participants’ identification number (1 to 14). The CSV files are named OAWXX-POSETTRACKER-CAMERA-WALK-YY.csv where XX is the participant number, POSETTRACKER is either Alphapose, Detectron, or OpenPose, CAMERA is either “top” or “bottom” for the top and bottom camera videos, respectively, WALK is either “front” and “back” which stand for front or back view walks, respectively, and YY is the number of walking bout. For example, the file “OAW01-Alphapose-bottom-back-1.csv” contains the first participant’s joint positions (in pixels) of the first back view walk captured by the bottom camera tracked by the Alphapose library. Finally, the videos folder contains the raw videos (.mp4 format) of each participants walk captured by the two cameras. The names of the video files are “OAWXX-top” or “OAWXX-bottom” where the additional “top” or “bottom” indicates the video recorded from the top or bottom camera, respectively. Participants’ faces are blurred in the videos to protect the identity of the participants. We also provided a Microsoft Excel file which contains the metadata with rows for participants and columns for participants’ demographic and clinical test scores.

## Technical Validation

**Fig. 2:**
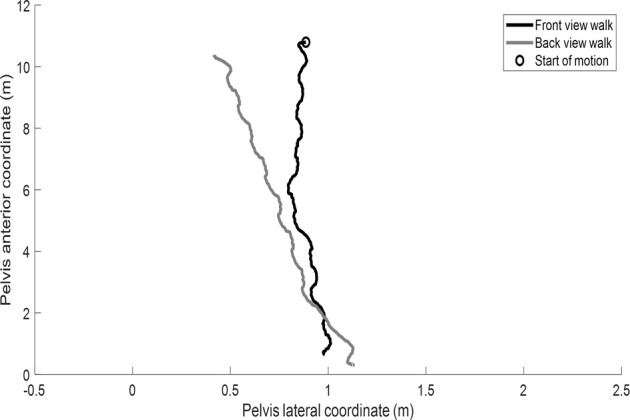
A representative participant’s pelvis motion in the horizontal plane for one front (black) and one back (grey) view. The circle shows the start of the front view walk.

**Fig. 3:**
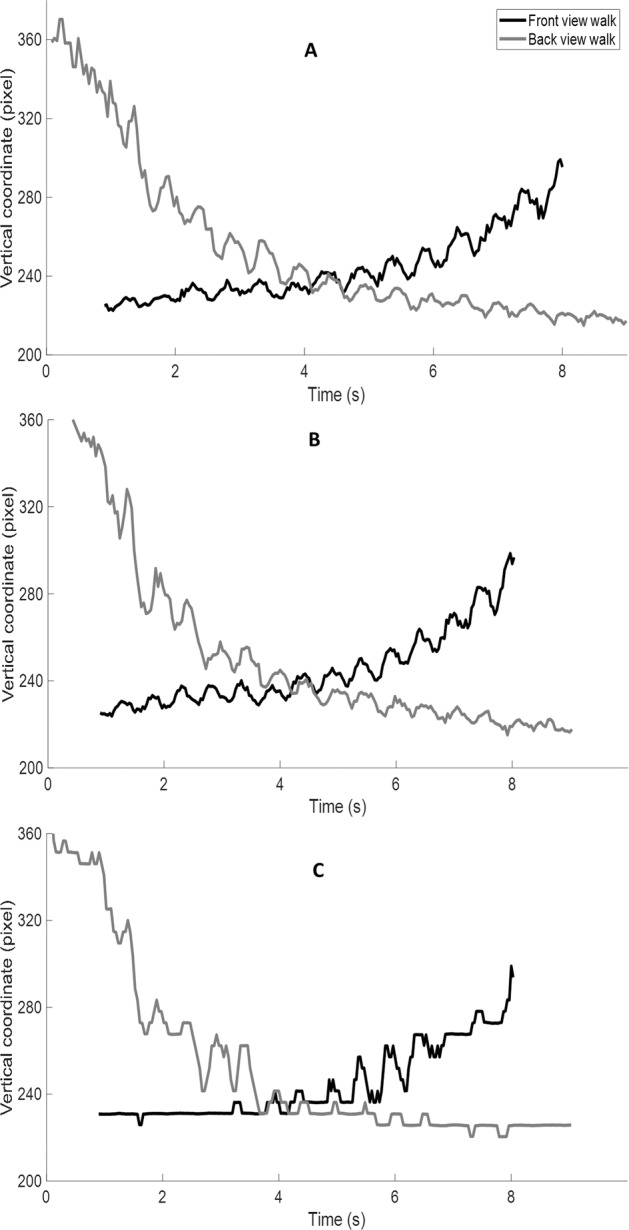
The head vertical coordinates (in pixels) of a participant’s front view (black) and back view (grey) walk tracked by (**A**) Alphapose, (**B**) Detectron, and (**C**) OpenPose.

The pelvis path in the horizontal plane of motion recorded with the Xsens system for a representative participants is shown in Fig. 2. Our initial data exploration revealed that due to drift problem common in the IMU systems^29,30^, the walking orientation in the motion capture system was shifted for some participants as can be seen in Fig. 2. This shift in the walking orientation, however, does not affect calculating the gait spatiotemporal variables (step length or width, for example) because gait variables are calculated as the distance between the body segments which is independent of walking orientation. The head vertical position (in pixels) of one front view and one back view walk for an example individual tracked with the three pose tracking algorithms is also depicted in Fig. 3. Note that the coordinate frame (x-axis is to the right and y-axis is downward) is located on the top left corner of the image (see Fig. 1) and a result when the participant walks away from the coordinate frame in the front view walks the pixel number increases (black-coloured graphs in Fig. 3). The opposite is true for the back view walks (grey-coloured graphs in Fig. 3) as can be seen in Fig. 3.

## Usage Notes

Our dataset has some limitations. First, the sample size of 14 participants is low, although it is in the range of studies on human walking. The initial plan was to recruit 20 older adults for this study. However, we had to stop data recording due to Covid-19 pandemic. Nevertheless, we were able to record a total of 373 and 241 steps for the front and back view walks, respectively, which is approximately equivalent to 34 and 22 steps per person for the front and back view walks, respectively (Table 2). This high number of recorded steps makes them suitable for statistical analysis and also for the training or fine-tuning of machine learning models. Another limitation of our data is the shift in orientation of the walking due the IMU drift problem^29,30^ although this shift of orientation does not affect the calculated gait variables.

To demonstrate an example use of our dataset, we calculated the correlation between the gait variables (calculated from both the 3D and 2D data) and the scores of the clinical gait and balance assessments. For the motion capture data, there were statistically significant correlations mainly between the temporal variables (cadence, step time, CV step time) and the clinical tests of POMA-balance, POMA-gait, and TUG, but not the between the spatial gait variables (step width, CV step width, and eMOS) and clinical gait and balance scores (Table 3). In particular, higher cadence was associated with better performances in POMA-balance and POMA-gait in both front (R = 0.60 and 0.67, respectively) and back view walks (R = 0.70 and 0.71, respectively) as well as better performance (shorter completion times) on the TUG test (R = −0.81 for front and R = −0.86 and back view walks). Similarly, lower step time was correlated with better POMA-balance (R = −0.64 and −0.73 for front and back walk views, respectively) and POMA-gait (R = −0.70 and −0.75 for front and back walk views, respectively) and longer TUG times (R = −0.85 and −0.90 for front and back walk views, respectively). Finally, a higher CV step time in front view was correlated with a lower POMA-balance score (R = −0.76) and a longer TUG time (R = 0.81). There was no correlation between the BBS and any of the gait variables.

**Table 3:** Correlation between the gait variables calculated using the inertial motion capture system and the clinical gait and balance scores.

		POMA-balance	POMA-gait	TUG	BBS
Cadence (steps per minute)	Front view	**0.60 (0.04)**	**0.67 (0.02)**	−**0.81 (<0.001)**	0.40 (0.23)
	Back view	**0.70 (0.01)**	**0.71 (0.01)**	**−0.86 (<0.001)**	0.48 (0.14)
Step time (s)	Front view	−**0.64 (0.03)**	−**0.70 (0.01)**	**0.85 (<0.001)**	−0.41 (0.21)
	Back view	−**0.73 (0.01)**	−**0.75 (0.01)**	**0.90 (<0.001)**	−0.47 (0.15)
Step width (m)	Front view	0.24 (0.47)	−0.25 (0.45)	−0.04 (0.91)	−0.09 (0.80)
	Back view	0.23 (0.49)	−0.13 (0.70)	−0.14 (0.67)	−0.21 (0.53)
CV step time	Front view	−**0.76 (0.005)**	−0.46 (0.14)	**0.81 (<0.001)**	−0.42 (0.20)
	Back view	−0.55 (0.07)	−0.13 (0.71)	0.51 (0.11)	−0.47 (0.14)
CV step width	Front view	−0.29 (0.37)	0.08 (0.79)	0.06 (0.86)	−0.01 (0.97)
	Back view	−0.52 (0.10)	−0.47 (0.15)	0.56 (0.07)	−0.09 (0.79)
eMOS (m)	Front view	0.21 (0.52)	−0.35 (0.28)	−0.05 (0.89)	−0.05 (0.89)
	Back view	0.17 (0.60)	−0.28 (0.40)	−0.10 (0.76)	−0.11 (0.75)

The values in the parentheses are the p-values. POMA = Tinneti performance-oriented mobility assessment; TUG = time up-and-go, BBS = Berg balance scale.

The trend of correlations between the gait variables calculated using the bottom video camera was similar to that of the Xsens system (Table 4). That is, higher POMA-balance and POMA-gait scores were correlated with higher cadence (R = 0.70 for POMA-balance in the back view walks, and R = 0.74 and 0.65 for POMA-gait with front and back view walks), and lower step time (R = −0.62 and −0.73 for POMA-balance and front and back view walks and R = −0.77 and −0.70 for POMA-gait and front and back view walks). Similarly, longer TUG times (lower speeds) were associated with a lower cadence (R = −0.70, and −0.87 for the front and back view walks, respectively) and a lower step time (R = 0.84 and 0.91 for the front and back view walks, respectively). Finally, in contrast to gait variables from the Xsens system, there were correlations between the BBS and some gait variables. In particular, higher BBS scores were associated with a lower step width (R = −0.63) and lower eMOS (R = −0.70) in the front view walks.

**Table 4:** Correlation between the gait variables calculated using the videos of the bottom camera and the clinical gait and balance scores.

		POMA-balance	POMA-gait	TUG	BBS
Cadence (steps per minute)	Front view	0.57 (0.07)	**0.74 (0.01)**	**−0.79 (<0.001)**	0.36 (0.27)
	Back view	**0.70 (0.02)**	**0.65 (0.03)**	**−0.87 (<0.001)**	0.44 (0.18)
Step time (s)	Front view	**−0.62 (0.04)**	**−0.77 (0.01)**	**0.84 (<0.001)**	**−**0.38 (0.24)
	Back view	**−0.73 (0.01)**	**−0.70 (0.02)**	**0.91 (<0.001)**	**−**0.44 (0.18)
Step width (m)	Front view	**−0.62 (0.04)**	**−**0.32 (0.34)	0.55 (0.08)	**−0.63 (0.04)**
	Back view	**−**0.51 (0.11)	**−**0.55 (0.08)	0.60 (0.05)	**−**0.52 (0.10)
CV step time	Front view	**−**0.05 (0.89)	**−**0.59 (0.05)	0.26 (0.44)	0.13 (0.70)
	Back view	0.22 (0.51)	**−**0.33 (0.32)	**−**0.11 (0.75)	0.15 (0.66)
CV step width	Front view	0.50 (0.12)	**−**0.08 (0.82)	**−**0.36 (0.28)	0.32 (0.33)
	Back view	0.33 (0.32)	**−**0.18 (0.60)	**−**0.02 (0.96)	0.41 (0.21)
MOS (m)	Front view	**−**0.57 (0.07)	**−**0.32 (0.34)	0.49 (0.13)	**−0.70 (0.02)**
	Back view	**−**0.51 (0.11)	**−**0.46 (0.15)	0.44 (0.18)	**−**0.60 (0.05)

The values in the parentheses are the p-values. POMA = Tinneti performance-oriented mobility assessment; TUG = time up-and-go, BBS = Berg balance scale.

The pattern of correlations for the top camera (Table 5) was similar to the bottom camera with some additions: a positive correlation (R = 0.74) between the CV step width in the back view walks and BBS, a negative correlation of −0.68 and −0.63 between the front view walks’ eMOS and POMA-balance, and between the back view walks’ eMOS and POMA-gait, respectively, and positive correlations of 0.73 between the TUG and eMOS in the front and back view walks.

**Table 5:** Correlation between the gait variables calculated using the videos of the top camera and the clinical gait and balance scores.

		POMA-balance	POMA-gait	TUG	BBS
Cadence (steps per minute)	Front view	0.58 (0.06)	**0.78 (<0.001)**	**−0.83 (<0.001)**	0.36 (0.28)
	Back view	**0.67 (0.03)**	**0.65 (0.03)**	**−0.86 (<0.001)**	0.38 (0.25)
Step time (s)	Front view	**−0.63 (0.04)**	**−0.81 (<0.001)**	**0.88 (<0.001)**	**−**0.38 (0.25)
	Back view	**−0.70 (0.02)**	**−0.70 (0.02)**	**0.90 (<0.001)**	**−**0.39 (0.24)
Step width (m)	Front view	**−**0.60 (0.05)	**−**0.36 (0.27)	0.58 (0.06)	**−0.69 (0.02)**
	Back view	**−**0.45 (0.16)	**−**0.59 (0.05)	0.55 (0.08)	**−**0.55 (0.08)
CV step time	Front view	0.32 (0.34)	**−**0.28 (0.41)	0.23 (0.50)	0.46 (0.15)
	Back view	0.03 (0.93)	0.04 (0.91)	0.00 (1.00)	**−**0.16 (0.64)
CV step width	Front view	0.44 (0.17)	**−**0.09 (0.79)	**−**0.25 (0.46)	0.42 (0.20)
	Back view	0.59 (0.05)	**−**0.12 (0.72)	**−**0.02 (0.95)	**0.74 (0.01)**
MOS (m)	Front view	**−0.68 (0.02)**	**−**0.41 (0.21)	**0.73 (0.01)**	**−**0.60 (0.05)
	Back view	**−**0.50 (0.11)	**−0.63 (0.04)**	**0.73 (0.01)**	**−**0.44 (0.17)

The values in the parentheses are the p-values. POMA = Tinneti performance-oriented mobility assessment; TUG = time up-and-go, BBS = Berg balance scale.

In all three modes of data capture, faster cadence and step times were associated with better performance on clinical gait and balance measures of POMA and the TUG (Tables 3–5). The opposite relationship exists between the step time and POMA and between the step time and TUG, i.e. higher step time is equivalent to lower speed (and a higher TUG) and lower gait quality (and thus a low POMA). Moreover, in the Xsens system only, there were correlations between the CV step time with POMA-balance (negative) and TUG (positive). These correlations, however, were not observed in the variability measured calculated from the cameras possibly, indicating that the vision-based pose tracking algorithms (Alphapose in our study) were not accurate enough to quantify gait spatiotemporal variability. This is consistent with our earlier findings with this dataset which showed there is a low correlation between spatial gait variables of the videos and the Xsens system^19^. Finally, there was no correlation between any of the temporal gait variables (cadence, step time, CV step time) in any of the recording modes and the BBS scores.

The correlation of spatial gait variables (step width, CV step width and eMOS) and clinical test scores was not consistent between the three recording modes. That is, while there was no correlation between the spatial gait variables and any of the clinical test scores in the motion capture system (Table 3), there were some correlations between the spatial gait variables calculated from the videos and the clinical scores. These correlations, however, did not show a consistent pattern (see the Results section for details). This finding also supports the results of our previous study^19^ based on this data where we demonstrated that there is a poor correlation between spatial gait variables of the videos and the Xsens system. This suggest that current video pose tracking algorithms are not accurate enough to calculate spatial gait variables from older adults and future studies should attempt to increase their pose tracking accuracy in this population.

One application can be improving the accuracy of the video-based pose tracking algorithms for the frontal plane videos of walking in older adults walking. This is important as previous datasets are mainly focused on sagittal plane videos of walking and in young adults. This will contribute to longitudinal monitoring of older adults’ walking living in the nursing homes using normal cameras and pose-tracking algorithms and thus a better fall risk assessment. Our Xsens and video datasets can also be used separately for a broad range of research purposes as they can be used for predicting gait events, calculating gait variables, and developing musculoskeletal models of walking.

## Data Availability

The files containing the codes for data analysis are available at Figshare.com^28^.
